# A Novel Beta-Defensin Antimicrobial Peptide in Atlantic Cod with Stimulatory Effect on Phagocytic Activity

**DOI:** 10.1371/journal.pone.0062302

**Published:** 2013-04-25

**Authors:** Jareeporn Ruangsri, Yoichiro Kitani, Viswanath Kiron, Jep Lokesh, Monica F. Brinchmann, Bård Ove Karlsen, Jorge M. O. Fernandes

**Affiliations:** Faculty of Biosciences and Aquaculture, University of Nordland, Bodø, Norway; California State University Fullerton, United States of America

## Abstract

A novel defensin antimicrobial peptide gene was identified in Atlantic cod, *Gadus morhua*. This three exon/two intron defensin gene codes for a peptide precursor consisting of two domains: a signal peptide of 26 amino acids and a mature peptide of 40 residues. The mature cod defensin has six conserved cysteine residues that form 1–5, 2–4 and 3–6 disulphide bridges. This pattern is typical of beta-defensins and this gene was therefore named cod beta-defensin (*defb*). The tertiary structure of Defb exhibits an α/β fold with one α helix and β_1_β_2_β_3_ sheets_._ RT-PCR analysis indicated that defb transcripts were present mainly in the swim bladder and peritoneum wall but could also be detected at moderate to low levels in skin, head- and excretory kidneys. In *situ* hybridisation revealed that *defb* was specifically expressed by cells located in the swim bladder submucosa and the oocytes. During embryonic development, *defb* gene transcripts were detectable from the golden eye stage onwards and their expression was restricted to the swim bladder and retina. *Defb* was differentially expressed in several tissues following antigenic challenge with *Vibrio anguillarum*, being up-regulated up to 25-fold in head kidney. Recombinant Defb displayed antibacterial activity, with a minimal inhibitory concentration of 0.4–0.8 µM and 25–50 µM against the Gram-(+) bacteria *Planococcus citreus* and *Micrococcus luteus*, respectively. In addition, Defb stimulated phagocytic activity of cod head kidney leucocytes *in* *vitro*. These findings imply that beta-defensins may play an important role in the innate immune response of Atlantic cod.

## Introduction

Defensins are important components of the innate arsenal of antimicrobial peptides (AMPs) and proteins (AMPPs) that provide protection against potential pathogens. These cationic AMPs with a β-sheet structure stabilised by disulphide bridges are widely distributed across both plant and animal kingdoms [1], [2]. In addition to their antimicrobial activity, animal defensins have other known biological roles. They are involved in host-microbiota interaction, immunomodulation through chemotaxis of immune cells and serve as potential links between innate and adaptive immunity [2], [3].

Bony fish are the most diverse group of vertebrates and they live in a complex aquatic environment, where they are exposed to water-borne pathogens and deeply affected by abiotic factors. In response, these animals secrete a wide range of AMPs and AMPPs as part of their defence mechanism [4], [5], [6], [7]. Even though defensins are important components of the immune system of multicellular organisms, their presence in teleosts have received scant attention. In fact, defensins have only been characterized from 8 teleost species to date. Fish defensins were first discovered through genome mining in model species, namely zebrafish *Danio rerio defb1* to *3* (*zfDB*-1 to -3), the green-spotted pufferfish, *Tetraodon nigroviridis defb1* and *2* (*tnDB*-1 and -2) and the tiger pufferfish, *Takifugu rubripes defb* (*fuDB*) [8]. Later, the presence of beta- defensin genes has been reported in other fishes: *defb1* to *4* isoforms (*omDB-*1 to -4) in rainbow trout, *Oncorhynchus mykiss* [9], [10], *defb* (*mkDB*) in medaka, *Oryzias latipes* [11], *defb* (*ogDB*) in orange-spotted grouper, *Epinephelus coioides* [12], *defb1* to *5* (*ofDBI-*1 to -5) in olive flounder, *Paralichthys olivaceus* [13], and most recently *defb* (*saDB*) in gilthead seabream, *Sparus aurata* [14]. Additionally, EST sequences that correspond to 3 beta-defensin isoforms in Atlantic salmon, *Salmo salar defb1*, -*3* and -*4* (*ssDB*-1, -3 and -4) and one beta-defensin in Nile tilapia, *Oreochromis niloticus defb* (*onDB*) were deposited in the National Center for Biotechnology Information (NCBI) database by Adzhubei et al. [15] and Lee et al. [16], respectively.

Constitutive beta-defensin expression varies between species and even among gene isoforms in the same species [8], [9], [13], [14], [17]. Furthermore, depending on the type of the tissue and or stimulatory factors, the genes are found to be either constitutive or inducible and they even have overlapping roles in protecting and maintaining fish health. Biological functions of beta-defensin have been investigated only in a few fish species and their multifunctional roles are still obscure. In most of the cases they were found to inhibit the growth of Gram - (**+**) and Gram - (**−**) bacteria [11], [13], [14], [17]. Others, like Defb of orange-spotted grouper and Defb1 of rainbow trout exhibited potential antiviral activity [10], [17]. In addition to its broad-spectrum antimicrobial activity, chemotactic ability has been reported for Defb from gilthead seabream [14]. Interestingly, defensins are thought to have some function related to the reproductive system in olive flounder and grouper [13], [17].

The Atlantic cod (*Gadus morhua*) is a marine cold-water fish species that is commercially and ecologically important. Its economic value as a farmed fish has stimulated interest in understanding its immune system due to the need to tackle diseases. The recent completion of its draft genome revealed that Atlantic cod has a peculiar immune system, inasmuch as it lacks MHC class II [18]. Moreover, antibody production against pathogens and or in response to vaccination is relatively poor [19], [20]. Hence, it is likely that Atlantic cod relies heavily on its innate immune system for host defence. In the present study we report the characterization of the first beta-defensin gene in Atlantic cod, which was termed *defb*. Our results demonstrated that this gene may play an important role in the cod innate immune response.

## Materials and Methods

### Naïve Fish – Husbandry and Tissue Sampling

Juvenile Atlantic cod were maintained indoors at the University of Nordland (UiN) Research Station, Bodø, Norway. The fish were reared at 7–8°C in 2,800 L fibre glass tanks that were part of a flow-through system and fed ad libitum with a commercial diet (Amber Neptune, Skretting AS, Norway). All animal handling protocols were in accordance with the guidelines adopted by the National Animal Research Authority (FDU) in Norway.

The transcription of *defb* gene was investigated in different tissues and organs of naïve specimens. Six randomly selected and apparently healthy fish weighing 200 to 300 g were anesthetised with 100 mg⋅L^−1^ of tricaine methanesulphonate (MS-222, Chemical Laboratories, Washington, USA) and immediately killed with a sharp blow to the head followed by transection of the spinal cord. Mucus samples were collected from the dorsal side of the body using a glass slide and then blood was collected from the caudal vein without anticoagulant, using a 1 mL syringe fitted with a 23 gauge needle. Thereafter, skin and fast muscle samples were taken from the left dorsal side of the fish. Next, the operculum was removed to excise the gill filament. Finally, following aseptic procedures internal organs and tissues - head kidney, excretory kidney, spleen, swim bladder, peritoneum wall, liver, pyloric caeca, proximal and distal intestines, rectum, heart and brain - were sampled, snap frozen in liquid nitrogen and stored at −80°C until use. Fractions of the same tissues and organs were fixed overnight in 4% paraformaldehyde prepared in phosphate buffered saline (0.1 M PBS, pH 7.4) treated with 0.1% diethylpyrocarbonate. Standard histological procedures were adopted to process these samples and embed them in paraffin.

### Immune Challenged Fish- Experimental Design, Husbandry and Sampling

The challenge experiment was conducted at the facilities of the Fish Health Unit of the Institute of Marine Research (HI), Bergen, Norway. Forty healthy unvaccinated Atlantic cod weighing around 60 g were randomly introduced into each of the three 500 L experimental tanks, which were part of a flow-through system. After the acclimation period, two fish from each tank were randomly sampled to collect the pre-challenge control samples. Skin samples from the left dorsal side of the fish, gill filament, head kidney and proximal intestine were collected adopting aseptic procedures, snap-frozen in liquid nitrogen and stored at −80°C until use. The challenge was performed using a *V. anguillarum* (strain H610 from the collection of the Fish Health Group at HI) cell suspension, prepared as detailed in Ruangsri et al [21]. Immediately prior to challenge, the water flow was stopped and the *V. anguillarum* suspension was added to each tank to attain a final concentration of 2.6×10^7^ cfu mL^−1^. Fish were exposed to bacteria for a period of one hour, after which the water flow was returned to normal. Following the challenge, two fish from each tank were sampled (6 fish per treatment) at 4 and 48 h to collect different tissues (post-challenge samples) as detailed above.

### Embryo Collection

Cod eggs were kindly supplied by CodFarmers ASA (Bodø, Norway). Unfertilised eggs were immediately frozen in liquid nitrogen and stored at −80°C until use. Artificially fertilised eggs from individual cod spawning pairs were incubated at 7°C without aeration in 5 L sterile glass bowls filled with 4 L drum-filtered (30 µm) UV-sterilised seawater. They were stocked at a density of approximately 10 mL eggs⋅L^−1^ and the bowls were covered with aluminium foil. Oxygen concentration in the incubating bowl was maintained above 6.5 mg⋅L^−1^ by replacing at least one third of the water daily. Embryos at different developmental stages (1-cell, 2-cells, 16-cells, oblong, germ ring, 50% epiboly, 10-somite and golden eye) and larvae (hindgut stage, first feeding and 20 days post-hatch) were observed under the optical microscope and approximately 50 specimens from each stage were collected, snap-frozen in liquid nitrogen and stored at −80°C for the *defb* gene expression study. In addition, embryos at the above developmental stages were fixed overnight in 4% paraformaldehyde, later dehydrated through graded levels of methanol and stored in 100% methanol at −80°C until used for whole mount *in situ* hybridisation.

### Database Mining of Cod Beta-defensin

The translated nucleotide sequences of defensins from different fish species were obtained from NCBI (http://www.ncbi.nlm.nih.gov/). These sequences were then used as queries to search the publicly available cod ESTs using TBLASTN. Possible defensin sequences retrieved were then used to design suitable primers for experimental confirmation.

### cDNA and gDNA Cloning of Cod Beta-defensin

cDNA sequences of beta-defensin were obtained from 20 days post-hatch whole larvae cDNA using the Def1 primer set shown on Table 1. Total RNA was extracted from approximately 50 to 100 mg of embryos using the Trizol method and cDNA synthesised using the QuantiTect RT kit (Qiagen GmbH, Hilden, Germany), as detailed in Fernandes et al. [22]. The amplification conditions were set as follows: initial denaturation at 95°C for 2 min, followed by 34 cycles of denaturation at 95°C for 30 sec, annealing step at 56°C for 30 sec, extension step at 72°C for 1 min and a final elongation at 72°C for 10 min. Amplicons were first separated by 1.2% (w/v) agarose gel electrophoresis, extracted from the gel, cloned and as detailed elsewhere [23].

**Table 1:** List of primers employed and their application in this study.

Primer	Sequence (5′–3′)	T (°C)	E (%)			Application	
Def1F	AGGATGTCTTGCCACCGAGTCT	56	−			Partial cDNA	
Def1R	AGATGGTGTCATTGCAGAGTCC					Probe synthesis	
Def2F	CAGTGCTGTTTAAGGATGTCTTG	58	−			Partial genomic	
Def2R	AGATGGTGTCATTGCAGAGTCC					DNA	
Def3F	TTTTGTTGAGAATGAGGCAGC	58	100			RT-PCR, qPCR	
Def3R	ATGAGACACACAGCACTGGAAT						
Def4F	TTCCCCTGGTCCTGCCCCAC	60		−	Recombinant		
Def4R	TTAAAAGAAATGAGACACACAGCAC			−	Defb expression		
Def5F	GTTCCCCTGGTCCTGCCCCAC	60		−	Recombinant		
Def5R	GGGATCCTTAAAAGAAATGAGACACACAGCAC			−	Defb expression		
eef1aF	CACTGAGGTGAAGTCCGTTG	58	91			q PCR	
eef1aR	GGGGTCGTTCTTGCTGTCT						
actbF	TGACCCTGAAGTACCCCATC	58	94			q PCR	
ActbR	TCTTCTCCCTGTTGGCTTTG						
rps9F	TCTTTGAAGGTAATGCTCTGTTGAGA	58	97			q PCR	
rps9R	CGAGGATGTAATCCAACTTCATCTT						
UbiF	GGCCGCAAAGATGCAGAT	58	97			q PCR	
UbiR	CTGGGCTCGACCTCAAGAGT						

T = annealing temperature; E = qPCR efficiency.

Genomic DNA (gDNA) was extracted from the spleen of *V. anguillarum* challenged fish using the Nexttec DNA isolation kit (Nexttec Biotechnologies GmbH, Leverkusen, Germany), following the manufacturer’s instructions. Partial gDNA sequence was obtained using the Def2 primer pair listed in Table 1 using a 6 min initial denaturation at 95°C and annealing at 58°C. PCR products were subsequently extracted, cloned and sequenced as above.

### Sequence Analysis

Following base calling, experimental sequencing data were assembled into contigs using the CodonCode aligner package (www.codoncode.com). Nucleotide sequences were translated with the ExPASy translation tool and basic properties of the mature peptides were predicted by the ProtParam software at ExPASy (www.expasy.ch). The presence of conserved domains and signal peptides was investigated with SignalP 3.0 (www.cbs.dtu.dk/services/SignalP), SMART (smart.embl-heidelberg.de) and ScanProsite (prosite.expasy.org) tools. Homology modelling was performed with the LOMETS server (zhanglab.ccmb.med.umich.edu/LOMETS) using the three-dimensional structure of the crotamine, a myotoxin from rattlesnake (*Crotalus durissus terrificus*) (PDB ID: 1H5O), as template. The results were then visualised with the DeepView, which also enabled the placement of the disulphide linkages (www.spdbv.vital-it.ch). Homologous sequences of fish beta-defensins were retrieved from the NCBI database using BLASTx or tBLASTx and the translated cod *defb* sequence as query. Putative beta-defensin peptide sequences were aligned with MUSCLE (www.ebi.ac.uk/Tools/msa/muscle/) and pair-wise sequence comparisons were performed with BioEdit [24].

Phylogenetic trees were constructed based on deduced full-length amino acid sequences using two different methods: i) the maximum likelihood (ML) algorithm implemented in PhyML (www.atgc-montpellier.fr/phyml/) with an LG model, estimated gamma shape parameter and an approximate likelihood-ratio test; and ii) Bayesian inference using a mixed model of amino acid substitution in MrBayes 3 [25] for 5×10^5^ generations and burning the first 500 trees. Accession numbers of the sequences used for homology comparisons and phylogenetic analysis are listed on Table 2.

**Table 2:** Beta-defensins from teleost fish available at the NCBI repository.

Species	Peptide	Accession number
*Gadus morhua*	Beta-defensin	JF733714
*Danio rerio*	Beta-defensin-like 1	CAJ57442
	Beta-defensin-like 2	CAJ57443
	Beta-defensin-like 3	CAJ57444
*Oncorhynchus mykiss*	Beta-defensin 1	CAK54950
	Beta-defensin 2	CAR82090
	Beta-defensin 3	CAR82091
	Beta-defensin 4	CAR82092
*Epinephelus coioides*	Beta-defensin	AAN02164
*Oryzias latipes*	Beta-defensin	ACG55699
*Paralichthys olivaceus*	Beta-defensin 1	ADA84138
	Beta-defensin 2	ADA84139
	Beta-defensin 3	ADA84140
	Beta-defensin 4	ADA84141
	Beta-defensin 5	ADA84142
*Sparus aurata*	Beta-defensin^a^	FM158209
*Tetraodon nigroviridis*	Beta-defensin 1	CAJ57644
	Beta-defensin 2	CAJ57645
*Takifugu rubripes*	Beta-defensin 1	CAJ57646
*Oreochromis niloticus*	Beta-defensin^a^	GR676330
*Salmo salar*	Beta-defensin 1^a^	CK892029
	Beta-defensin 3^a^	CK895520
	Beta-defensin 4^a^	EG781611

a Predicted from EST sequences.

### Expression of Cod Beta-defensin

#### 1. Semi-quantitative RT-PCR

Total RNA was extracted from approximately 100 mg of sample using the Trizol method and cDNA synthesised using the QuantiTect RT kit (Qiagen), as detailed in Fernandes et al. [22]. RNA quality and quantity were evaluated by 1.2% agarose gel electrophoresis and spectrophotometry using the Quant-iT RNA assay kit (Qubit Fluorometer, Invitrogen, California, USA). Cod beta-defensin was amplified with the Def3 primer pair (Table 1) and beta-actin (*actb*) was used as an endogenous control. Amplification conditions were set as follows: initial denaturation at 95°C for 2 min, 30 cycles of denaturation at 95°C for 30 sec, annealing at 58°C for 30 sec and extension at 72°C for 1 min, followed by final elongation at 72°C for 10 min. Amplicons were separated by electrophoresis on a 1.2% (w/v) agarose gel, photographed and analysed using the ChemiDoc gel documentation system from BioRad (Laboratories Inc, California, USA).

#### 2. Whole-mount and section *in situ* hybridisation

Cod beta-defensin was subcloned using the Def1 primer pair and a DNA template for probe synthesis was obtained from the corresponding pCR4-TOPO plasmids by PCR using standard M13 primers (Invitrogen) and the thermocycling conditions described in section 2.5. T7 and T3 RNA polymerases (Roche, East Sussex, UK) were used to synthesise digoxigenin (DIG)-labelled RNA probes by *in vitro* transcription, according to the manufacturer’s protocol.

For each selected developmental stage (i.e. bladder, hindgut and first feeding stage), five cod embryos were used. Paraffin-embedded tissue samples of naïve fish were used to prepare 4–5 µm microtome sections (Shandon Finesse, Thermo Scientific, Barrington, USA) on poly-L-lysine coated slides (VWR International, Leuven, Belgium). The slides were then incubated overnight at 50°C, dewaxed by two subsequent treatments with xylene and rehydrated through decreasing graded levels of methanol. Embryos and tissue sections were permeabilised with 20·g⋅mL^–1^ proteinase K (Roche) for 20 min and 30 min, respectively. *In situ* hybridisation (ISH) was performed essentially as described previously in Fernandes et al. [23]. Sense probes were used as negative controls. Tissues sections were counterstained with neutral fast red (Sigma, Missouri, USA). Whole embryos and tissues were then observed under a binocular microscope (Leica DM4000B, Leica Microsystem, Wetzlar, Germany) and a Lieca DMIL microscope (Leica), respectively, and images were acquired with a Leica DFC420 camera (Leica).

#### 3. Quantitative real-time PCR (qPCR)

Quantification of cod beta-defensin gene in 10× diluted cDNA samples was carried out in duplicate using ubiquitin (*ubi*) and elongation factor 1a (*eef1a*) for proximal intestine or ribosomal protein s9 (*rps9*) and *eef1a* for head kidney, gill and skin as reference genes. These reference genes had been shown to have stable expression levels in the particular tissues from our studies [26]. Primers were designed across intron/exon borders to prevent amplification of contaminating genomic DNA (Table 1). Real-time PCR (qPCR) was performed using a StepOne Plus Real-Time PCR System (Applied Biosystems) with SYBR Green chemistry (Power SYBR Green PCR Master Mix, Applied Biosystems). No-template and minus reverse transcriptase controls were included for each primer pair. The thermal profile for qPCR was 50°C for 2 min and 95°C for 10 min, followed by 40 cycles of 95°C for 15 sec, 58°C for 20 sec and 72°C for 30 sec. The specificity of PCR amplifications was determined by melting curve analysis, run at the temperature range 60–90°C with a ramp speed of 0.5°C, and further confirmed by Sanger sequencing.

Five-fold dilutions (1∶1 to 1∶625) of pooled RNA were used to make standard curves to determine amplification efficiencies (E) and raw transcript amounts. PCR efficiencies were calculated using the equation E = (10^−1/m^ − 1)·100, where *m* is the slope of the linear regression model fitted over log-transformed data of the input cDNA concentrations *versus* C_T_ values [23]. Relative beta-defensin transcript levels was normalised against expression of the reference genes using the geometric normalisation factor determined by GeNorm [27], as detailed elsewhere [28]. The effects of both feed groups and time after challenge on the expression of *defb* in each tissue were analysed by two-way ANOVA (Graph pad Prism Version 5.03), after confirming that data had equal variance and were normally distributed.

### Recombinant Expression and Purification of Cod Beta-defensin

The DNA sequence corresponding to the mature cod Defb was amplified by PCR using the primers Def4F and Def4R (Table 1). A second amplification round was then performed to adjust the frame in the expression vector using the primers Def5F, which added one guanine residue at 5′ end, and Def5R, which added a BamH I restriction site immediately after the stop codon (Table 1). All PCRs were performed with a high fidelity PCR enzyme (Phire Hot Start II, Thermo Fisher Scientific Inc., Waltham, USA). The insert was cloned into the expression vector pET-44a(+) using T4 DNA ligase (Invitrogen) after digestion with PshA I (New England Biolabs, Ipswich, MA, USA) and BamH I (Invitrogen).

The above pET-44a(+)-Defb plasmid vector was used to transform Rosetta-gami B (DE3) pLysS (Merck KGaA, Darmstadt, Germany) *Escherichia coli*. The recombinant protein was overexpressed by induction with 0.3 mM IPTG, extracted using the BugBuster protein extraction solution (Novagen/Merck) and purified with the ProBond purification system (Invitrogen), according to the manufacturer’s instructions. ProBond purified fractions were pooled and dialysed against 1% Tween 20, 1 mM CaCl_2_, 50 mM Tris-HCl (pH 8.0) using a dialysis membrane (MWCO 3.5 kDa) for 16 hours at 4°C. The recombinant mature cod Defb was cleaved from the fusion protein by digestion with 0.1 unit·mL^−1^ of EK Max enterokinase (Invitrogen) for 16 hours at 25°C, filtered through an ultrafiltration spin column (10 kDa cut-off) and purified by solid phase extraction with SepPak C_18_ cartridges (Waters, Milford, MA, USA). The mature peptide fraction was eluted with 90% (v/v) methanol, dried and resuspended in 0.01% (v/v) acetic acid.

### MS Analysis of Recombinant Beta-defensin

The 4.5 kDa band from a SYPRO ruby stained gel was excised, in-gel reduced, alkylated and trypsinised as described elsewhere [29]. It was then subjected to LC-MS/MS analysis (ESI Quad TOF, Micromass/Waters, MA, USA). Peak lists (pkl) were generated by the Protein Lynx Global server software (version 2.1, Micromass/Waters, MA, USA). The resulting pkl files were adjusted with an internal trypsin standard and used for protein identification with the FindPept tool (hweb.expasy.org/findpept/) based on monoisotopic masses with oxidised methionines and cysteines converted to their carbamidomethyl derivative.

### Bioactivity of Cod Beta-defensin

#### 1. Antibacterial activity

Antibacterial activity was measured by a liquid growth inhibition assay in a 96 well-microtitre plate, as reported [30]. The following 4 bacterial strains were tested: *Planococcus citreus*, *Micrococcus luteus*, *Aeromonas salmonicida* and *Vibrio anguillarum*. A mixture of 50 µL of defensin (0.1 to 50 µM dilution series) and 50 µL of bacterial suspension (1×10^5^ colony forming unit/mL) in Muller–Hinton broth (Difco Laboratories, Detroit, MI, USA), was incubated at a suitable temperature until log phase was reached for each bacterium (Table 3). After incubation, bacterial growth was quantified by absorbance measurement at 595 nm using a microtitre plate reader (FLUOstar Optima, BMG Labtech GmbH, Ortenberg, Germany). The minimal inhibitory concentration was defined as concentration of defensin that inhibited growth by 50% compared to the control without peptide.

**Table 3:** Antibacterial activity of recombinant cod beta-defensin.

	Gram	T	MIC^a^	
		(°C)	(µM)	(µg·ml^−1^)
*Micrococcus luteus* ATCC 4698	+	37	25–50	125–250
*Planococcus citreus* NCIMB 1493	+	25	0.4–0.8	2–4
*Aeromonas salmonicida* NCIMB 1102	−	20	>50	>250
*Vibrio anguillarum* NCIMB 2133	−	20	>50	>250

a The minimal inhibitory concentration (MIC) is defined as the concentration of peptide that inhibits bacterial growth by 50% compared to the negative control without peptide.

#### 2. Phagocytosis assay

Head kidney leucocytes from five Atlantic cod were prepared according to Meng et al. [31] and suspended in L-15 medium. All solutions were adjusted to an osmolality of 380 mOsm with NaCl. Phagocytic activity was measured using pHrodo BioParticles (*E. coli* bioparticles, Invitrogen). The cell suspension (10^6^ cells in 100 µL) was placed in wells of a flat bottom 96-well plate and incubated for 2 hours at 15°C for cell attachment. The media was replaced with L-15 with 2% fetal calf serum and the cells were kept overnight (14 h) at 15°C. The cell culture medium was then aspirated to remove cell debris and unattached cells, washed with Hanks buffer supplemented with NaCl and replaced with working solution of the same buffer but with pHrodo bioparticles and recombinant cod Defb (0.2 to 20 µM). In addition, an aliquot of the particles was opsonized with 5% cod serum at 4°C for 1 hour and added to separate wells with cells. Cell viability, assessed in separate wells, was more than 90%, as determined by the trypan blue exclusion method. Duplicate samples for each fish were treated and measured in parallel. After incubation at 15°C for 4 hours, fluorescence was measured in a microplate reader (FLUOstar Optima, BMG Labtech) with excitation at 560 nm and emission at 590 nm. The effect of cod Defb on phagocytic activity was determined according to the following equation: % effect = (I_test_ − I_nc_)/(I_op_ − I_nc_)·100, where I_test_, I_nc_ and I_op_ are the mean fluorescent intensities of the test group, non-cell control and opsonized positive control, respectively. Data from the phagocytosis assay were analysed by paired Student’s t-test or Mann-Whitney U test when the data were not normally distributed.

## Results

### Sequence Analysis of Cod Beta-defensin

**Figure 1:**
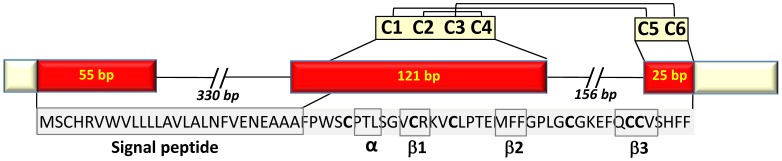
Schematic representation of the beta-defensin gene in Atlantic cod. Pale yellow boxes represent partial 5′ and 3′untranslated regions. Red boxes indicate the coding region corresponding to the 66 amino acid residue peptide precursor. The predicted signal peptide and important secondary structure elements in the mature peptide are boxed. Conserved cysteine residues are highlighted in bold letters and predicted disulphide linkage patterns are also shown.

After identifying two cod ESTs with homology to fish defensins, specific primers were design to amplify the complete defensin coding sequence (CDS) in Atlantic cod. The cDNA sequence obtained was 248 nucleotides long and comprised a partial 3 base pair (bp) 5′-untranslated region (UTR), a 201 bp open reading frame (ORF) that encoded the 66-residue peptide precursor, and a partial 44 bp 3′UTR. Sequence homology searches against the non-redundant protein database at NCBI revealed that cod defensin is most similar to fuDB-1, a defensin-like protein 1 in tiger pufferfish (89%; E-value = 10^−20^), and omDB-1, a beta-defensin 1 from rainbow trout (85%; E-value = 10^−18^). Hence, the cod defensin sequence was named as beta- defensin (Defb*)* and deposited in GenBank under the accession JF733714. The cod *defb* was also partially sequenced and revealed a CDS of three exons and two introns, similarly to other fish beta-defensin members (Fig. 1).

**Figure 2:**
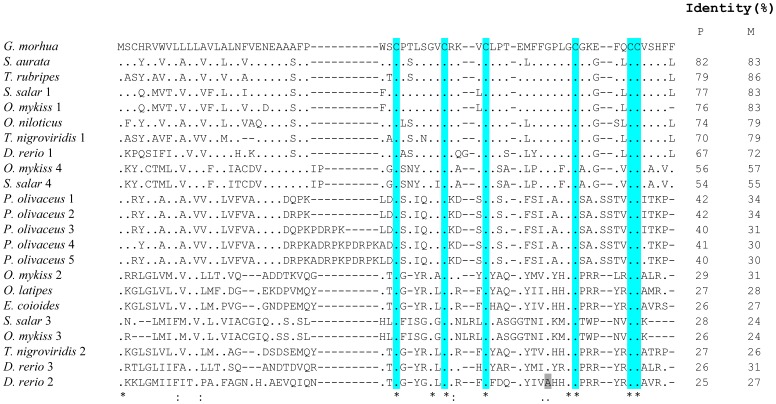
Multiple alignment of cod beta-defensin amino acid sequences with other beta-defensin proteins from teleosts. Identical amino acid to cod beta-defensin are represented by dots, while identity, similarity and weak similarity residues across fish taxa are represented by (∗), (:) and (⋅), respectively. The six conservative cysteine residues are shaded in blue. Identity between cod beta-defensin of various beta-defensin peptide precursors (P) or mature peptides (M) are also shown on the right. Accession number of the sequences are listed on Table 2.

The 66-amino acid cod Defb precursor contained an N-terminal signal peptide with a likely cleavage site between amino acid Ala 26 and Phe 27 (AAA_FP), as well as the C-terminal beta-defensin signature. The signal peptide is encoded by the first exon and part of the second exon, whilst the mature peptide corresponds to part of the second and third exons. The latter has a calculated MW of 4.49 KDa, a predicted *pI* of 7.79 and an overall net charge of +1. Defb has six conserved cysteine residues at positions 31, 38, 42, 54, 60 and 61 in the mature peptide, and an additional 3 conserved glycines at positions 36, 50 and 53. Cysteines C1, C2, C3 and C4 are located in the second exon, while C5 and C6 are found in the last exon (Fig. 1). The putative amino acid sequence of Defb was compared to 22 beta-defensins from 10 fish species (Table 2). Sequence alignments showed that cod Defb has a conservative motif of common beta-defensins, sharing six conserved cysteines and additional 3 glycine residues that exist close to the positions of C2 and C4 in most species, the only exception being zebrafish Defb2, where the glycine at position 50 is replaced by alanine (Fig. 2). The identity at the protein level between cod Defb peptide precursor and their homologues from fish species varied widely, ranging from 25 (Defb2 of zebrafish) to 82% (Defb of gilthead seabream).

### The Structure of Cod Defb

**Figure 3:**
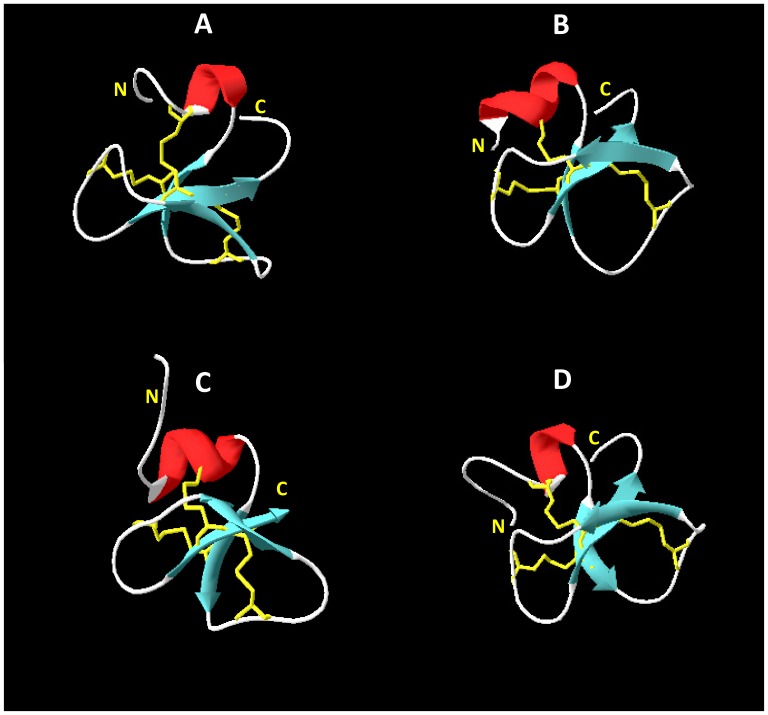
Three dimensional structures of cod Defb (A), crotamine (B), mBD8 (C) and zebrafish Defb1 (D). The presence of an α-helix (red), three antiparallel β-strands (blue), and the disulphide linkages of 1–5, 2–4 and 3–6 pattern (yellow) were found in all species. Homology modelling of cod Defb was performed using the PBD structure of crotamine, a neurotoxin from rattlesnake, *Crotalus durissus trrificus* (PDB ID: 1H5O), and mouse beta-defensin 8 (mBD8, PDB ID: 1E4R) as templates.

The predicted three dimensional structure of cod Defb is shown in Fig. 3. Comparison of the cod Defb peptide signature with the structure of crotamine revealed that Defb contains a short N-terminal α-helix and a small antiparallel triple-stranded β-sheet arranged in an αβ1β2β3 topology (Fig. 3A, B). Compared to other beta-defensins, cod Defb has the highest structure similarity with that of mouse mBD8 (Fig. 3C). Amongst fish beta-defensins, cod Defb structure resembles zebrafish Defb1, with 72.1% identity between both sequences (Fig. 3D). Disulphide linkages of each beta-defensin were examined using Swiss-PdbViewer and all bridges were predicted to exist between cysteine residues 1–5, 2–4 and 3–6, in common to all other vertebrate beta-defensins (Figs. 1, 3).

### Phylogeny of Cod Beta-defensin

**Figure 4:**
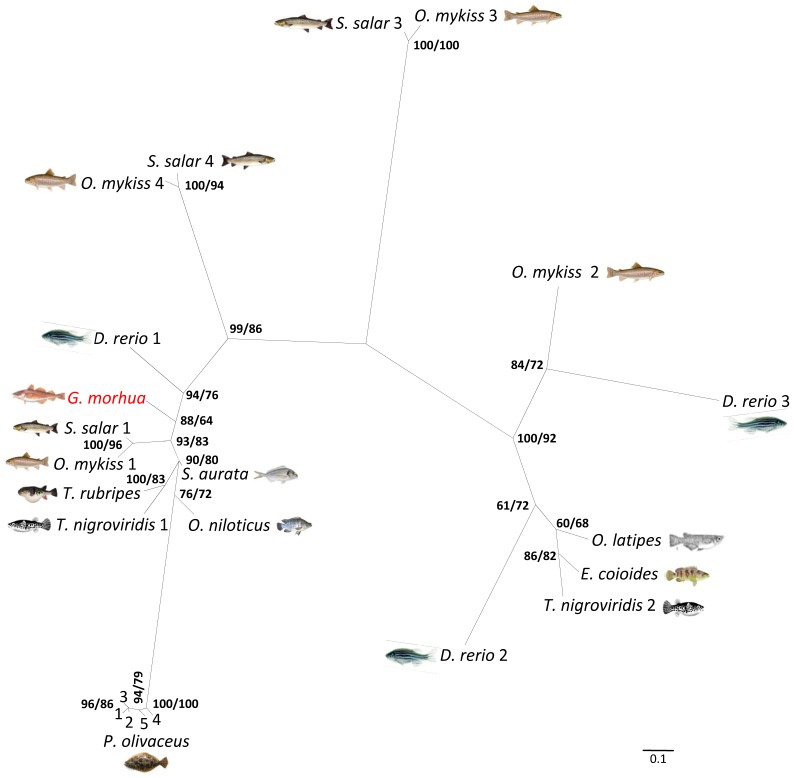
Unrooted radiation tree illustrating the phylogenetic relationship between fish beta-defensins. Cod beta-defensin (highlighted by red) falls into the same clade with a large group of other beta-defensins from different teleost taxa. Bayesian posterior probabilities and maximum likelihood values are indicated as percentages on the tree nodes, respectively.

Bayesian and likelihood phylogenetic reconstructions produced trees with same topologies that consisted for two main clades (Fig. 4). The largest cluster included cichlidae, sparidae, all group 1 beta-defensins from salmonidae, tetraodontidae, cyprinidae and beta-defensin-1 to -5 from paralichthyidae. This clade followed the accepted taxonomic relationship between these teleost species and cod Defb (highlighted in red) is placed between cyprinid and salmonid defensins, as expected. The second group comprised beta-defensins from salmonidae, tetraodontidae and cyprinidae, adrianichthyidae and serranidae. Salmonidae beta-defensins 3 and 4 form two monophyletic groups.

### Developmental Expression Pattern and Tissue Distribution

**Figure 5:**
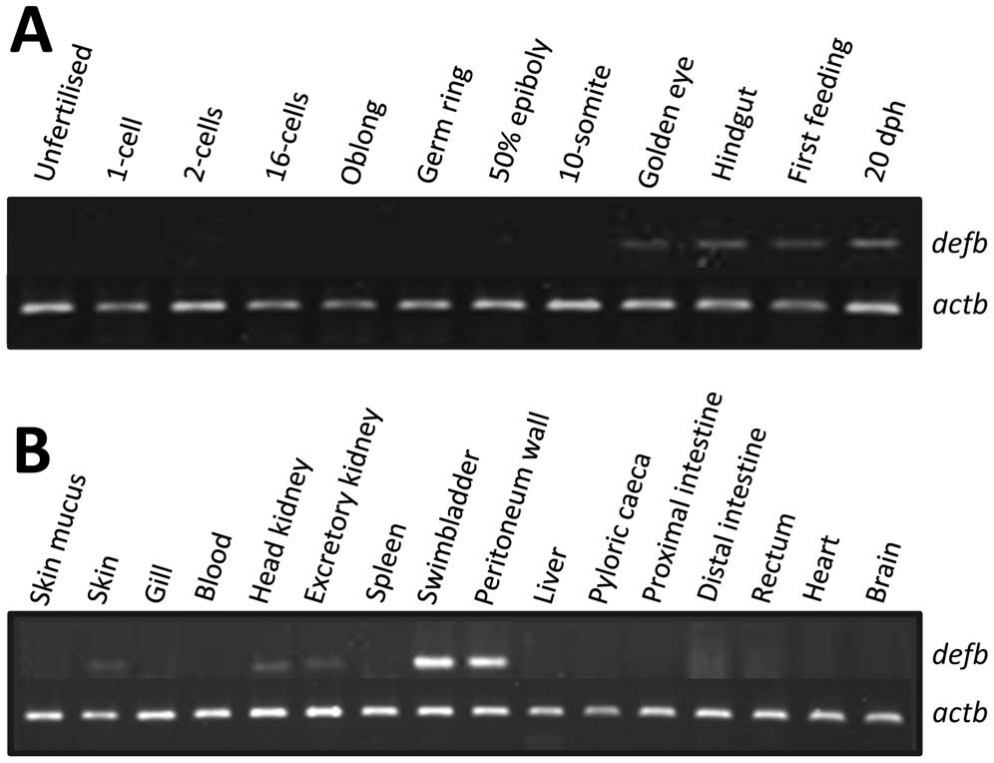
Representative expression pattern of *defb* (A) during embryonic development and (B) in various juvenile cod tissues, as determined by semi-quantitative RT-PCR. Beta-actin (*actb*) was used as a reference gene. Amplicon sizes are 139 bp and 175 bp for *defb* and *actb*, respectively.

**Figure 6:**
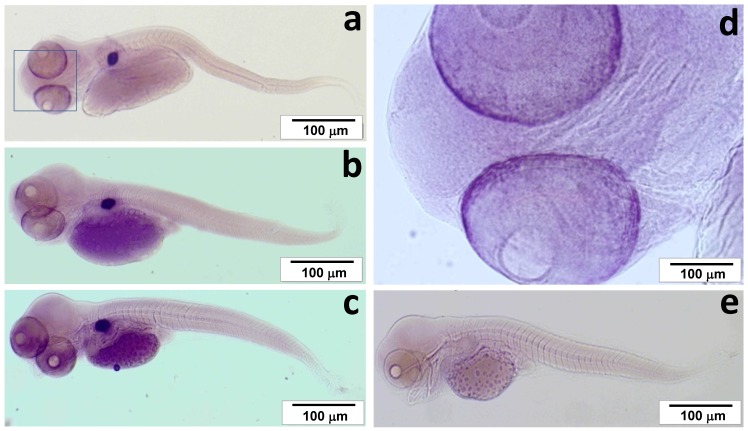
Expression of *defb* in bladder (a), hindgut (b) and first feeding (c) cod larval stages. *Defb* is highly expressed in the swim bladder and in the eye. The eye is magnified showing a strong positive signal on the edges of the retina (d). No positive signal is seen in negative controls with the sense probe (e).

**Figure 7:**
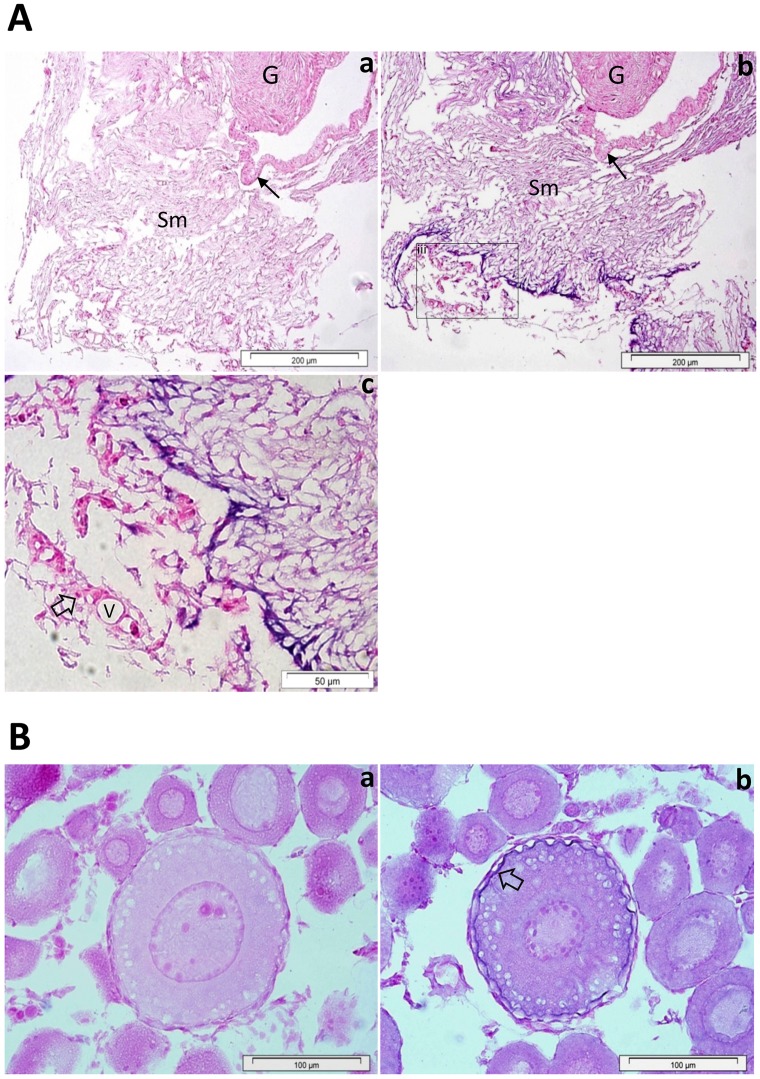
Localization of *defb* in the swim bladder (A) and oocytes (B) of Atlantic cod. In the swim bladder of juvenile fish, *defb* is expressed in the loose connective tissue of the submucosa (Sm) (Ab) but not in the secretory epithelial cells of the gas gland (G) and the epithelium of swim bladder wall (arrow). The sense mRNA probe (a) shows no *defb* positive signal (Aa). A portion of the swim bladder’s submucosa is magnified, (Ac) showing a strong *defb* positive signal in the loose connective tissue but not in red blood cells (open arrow) or blood vessels (V). In oocytes (B), *defb* transcripts are present along the egg membrane of early vitellogenic stage (Bb, open arrow). No signal was observed with the negative control probe (Ba).

During embryonic development *defb* transcription was found from the golden eye stage (approximately 225 h post fertilization) onwards (Fig. 5A). In naïve juvenile cod, *defb* had a restricted expression pattern amongst the 16 tissues examined (Fig. 5B). It was expressed mainly in the swim bladder and peritoneum wall but also detected at moderate to low levels in skin, head and excretory (posterior) kidneys. A more detailed analysis by *in situ* hybridisation revealed that *defb* is expressed in the developing swim bladder and retina of cod embryos at bladder and hindgut stages, as well as in first feeding larvae (Fig. 6). In juvenile fish, there was a strong *defb* signal, located in the loose connective tissue of the submucosa (Fig. 7A). *Defb* transcripts were not detectable in most of tissues tested, namely gills, head and excretory kidneys, skin, spleen, liver, pyloric caeca, intestine and muscle. Notably, *defb* was present in oocytes, particularly in an early vitellogenic stage, where a strong signal was seen along the membrane of the egg (Fig. 7B). No *defb* mRNA signal was observed in the control samples (sense mRNA probe) of tissue sections, embryos or larvae.

### Induction of Cod Defb by Antigenic Challenge

**Figure 8:**
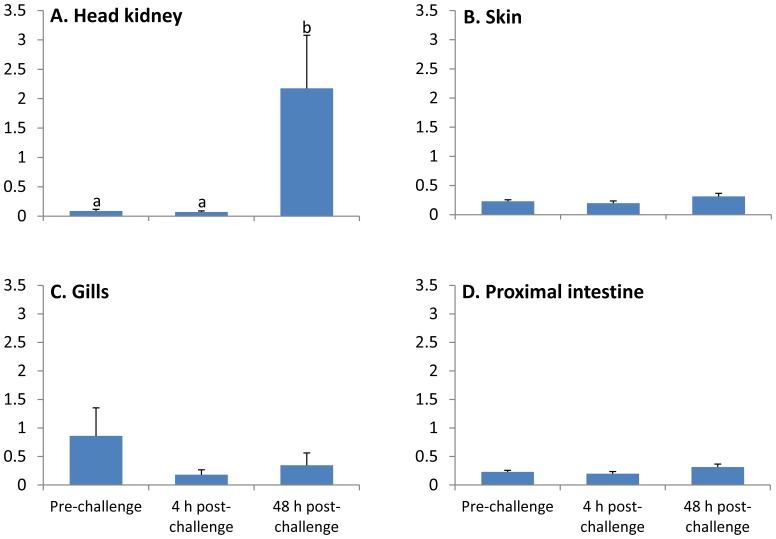
Relative expression of *defb* in immune-related tissues of Atlantic cod upon challenge with pathogenic bacteria (*Vibrio anguillarum*, strain H610). Each bar represents the mean (n = 6) with error bars indicating the SEM. Different letters above the bar indicate statistically significant expression differences between the challenged group of a particular treatment. Data are normalised against expression of *ubi* and *eef1a* for proximal intestine or *rps9* and *eef1a* for head kidney, skin and gill.

Relative expression of *defb* in each tissue tested was normalised against two reference genes that were found to be stable in the tissues tested (see section 2.7.3). Significantly higher *defb* expression was observed in the head kidney at 48 h post-challenge compared to 4 h post-challenge (30.6-fold, p<0.01) and the pre-challenge group (24.8-fold, p<0.01) (Fig. 8A). In skin and proximal intestine, *defb* transcript levels remained unchanged by challenge with *V. anguillarum* (Fig. 8B, D). An apparent down-regulation of *defb* was observed in the gills at 4 and 48 hours post-challenge, albeit this difference was not significant (Fig. 8C, p>0.05). Overall, there was a significant interaction between time and relative *defb* expression levels in all tissues tested (p = 0.006).

### Bioactivity of Recombinant Cod Beta-defensin

The recombinant cod Defb was highly expressed in *E. Coli* RosettaGami (DE3) cells as a 66 kDa fusion protein with a Hisx6-Nus Tag at the N terminus. After enterokinase digestion, the mature 4.5 kDa Defb was released from the fusion protein (Supplementary Fig. S1). Identity of the recombinant mature cod defensin was confirmed by mass spectrometry, since the identified peptide fragments covered the entire sequence (Supplementary Table S1).

Cod Defb was active against Gram-(+) bacteria, with minimal inhibitory concentrations against *P. citreus* and *M. luteus* in the range 0.4–0.8 µM and 25–50 µM, respectively. In contrast, no antibacterial activity was detected against the Gram-(**−**) bacteria tested, i.e. *A. salmonicida* and *V. anguillarum* (Table 3).

**Figure 9:**
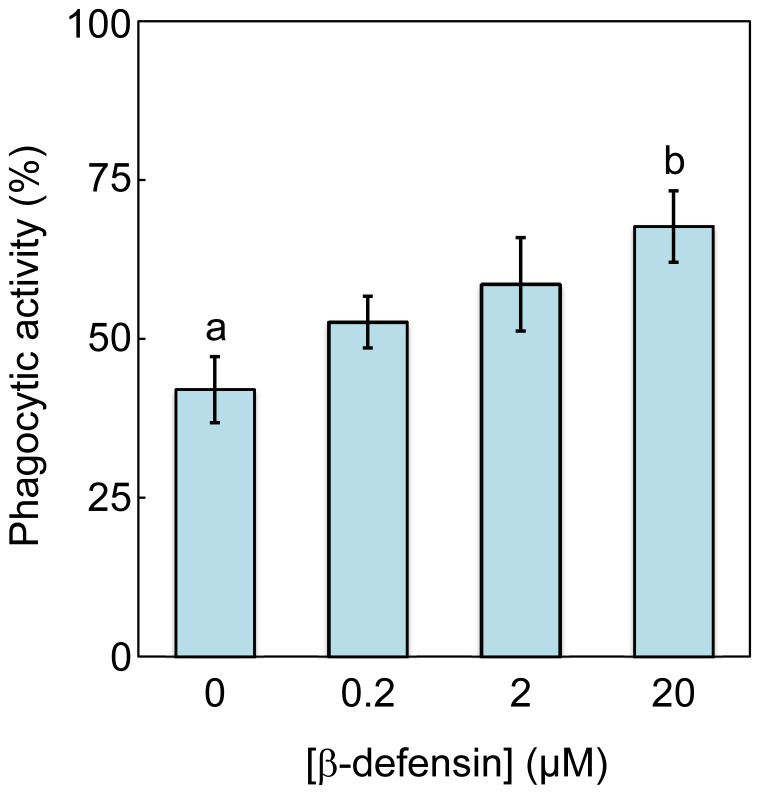
Effect of cod beta-defensin on phagocytic activity of cod head kidney leucocytes. Data are represented as mean ± SEM (n = 5) and different letters indicates significant between two groups (p<0.05).

The phagocytic activity of cod head kidney leucocytes was stimulated by cod Defb in a concentration-dependent manner (Fig. 9). At a concentration of 20 µM cod Defb the phagocytic index was 68±5.63% (mean ± SEM, n = 5), which corresponded to an increase of 62% compared to the control without defensin (p<0.05, Fig. 9).

## Discussion

In the present study, we report for the first time the identification and characterization of a defensin gene in a gadoid fish. The putative cod defensin precursor comprises a carboxy-terminal signal peptide and an amino-terminal defensin signature with six conserved cysteine residues that form 1–5, 2–4 and 3–6 disulphide linkages. This bridge pattern along with the short spacing between C1 and C2 indicate that the cod defensin is a member of the vertebrate beta-defensin family rather than an alpha-defensin [8], [32] and, therefore, it was named cod beta-defensin or Defb for short.

Cod *defb* and beta-defensin genes of zebrafish, tiger pufferfish, medaka, olive flounder, rainbow trout and orange-spotted grouper share a similar structure, consisting of three exons and two introns. Moreover, cysteine residues C1 to C4 are located in exon 2, whereas C5 and C6 are in exon 3 [8], [9], [11], [13], [17]. The putative mature cod Defb has 4.49 kDa, a net charge of +1 and a predicted isoelectric point of 7.79, features that are typical of fish defensins. Our phylogenetic analysis placed cod Defb between zebrafish and salmonid type 1 defensins, in accordance to currently accepted taxonomic relationships between these taxa. A high degree of conservation between known structural elements was observed between cod and other fish defensins. The six cysteine residues also present in cod defensin are conserved across human defensins (both alpha and beta), chicken beta-defensins and snake crotamine [8], which indicates that they are essential for the structure and function of these molecules. In human, the hBD2 and hBD3 exhibits an α/β fold, with an α helix and β_1_β_2_β_3_ sheets with a 1–5, 2–4 and 3–6 disulphide linkage pattern [2]. A similar structure is seen for cod Defb, even if it is more akin to those of crotamine of rattlesnake and mBD8 of mouse. This could be explained by the presence of a conserved motif Gly-X-Cys in the second β-strand in humans, in contrast to the first β-strand in all fish taxa, snake and mouse. Amongst fish species, the cod Defb tertiary structure has greater similarity to the predicted structure of zebrafish Defb1 reported by Zou et al. [8]. Beta-defensins from several fish species (e.g., the gilthead seabream, rainbow trout, grouper, medaka), that are homologous to cod beta-defensin are known to exhibit antimicrobial activity [10], [11], [14], [17]. In addition, a wide spectrum of biological functions of crotamine and mBD8 has been reported, including immune modulation and chemotactic stimulation [33], [34]. The structural similarity between cod Defb and these other defensins indicates that cod defensin is likely to have at least some of the aforementioned biological properties.

Generally, the distribution of fish beta-defensin genes in tissues varies between different fish species and even between gene isoforms within the same species. For instance, the *defb1* and *defb3* of zebrafish, all the gene paralogues (*defb1* to *4*) of rainbow trout and the *defb* of gilthead seambream were expressed in a wide range of tissues and organs [8], [9], [14]. On the other hand, the *defb* of orange spotted grouper was found to be expressed only in pituitary and testis, and the expression of *defb2* gene of zebrafish was restricted to the gut tissue [8], [17]. We had previously demonstrated that a wide range of tissues and organs of Atlantic cod have active antimicrobial factors [21], including the two piscidin peptides, which can inhibit various strains of bacteria [22], [35]. In the present study we show that even in naïve juvenile cod, *defb* is expressed at low levels in several immune-related organs, including the skin, peritoneum wall and kidneys, indicating that *defb* may be an important component of the innate immune system of Atlantic cod.

High expression levels of *defb* gene were found in the swim bladder of cod. Furthermore, *in situ* hybridisation revealed that this gene was expressed in the developing swim bladder in larvae and in the loose connective tissue of the submucosa juvenile fish. Also in zebrafish, *defb1* is present in the same organ of larvae, juveniles and adult fish [36]. The teleost swim bladder is an embryological derivative of the gastrointestinal canal and its wall is morphologically similar to the gut wall [37] and its known functions are associated with buoyancy, respiration and communication [38]. It is plausible that the swim bladder plays a role in host defence, since this organ is homologous to the mammalian lung [39], which is known to express several beta-defensins [40]. It is also worth to investigate whether cod beta-defensin or piscidins have significant roles in hydrostatic regulation. In mammals, beta-defensins are known to be involved in protection and tissue repair [41], [42]. A previous study on medaka reported that beta-defensin was abundantly present in several parts of eyes and the authors suggested that these molecules may hold therapeutic potential [11]. Moreover, levels of beta-defensin mRNA in the eye increased by more than 10-fold following stimulation with lipopolysaccharide for 12 h [11]. The presence of *defb* transcripts in the retina of cod larvae might be explained by their bioactivity against pathogens present in the complex aquatic environment or by a developmental role for defensins.

Beta-defensins that are expressed in the reproductive tract of several higher vertebrate species are thought to protect the sperm and the egg from microbial infection, enabling them to survive in the genital tracts [2], [43]. *Defb* of orange spotted grouper and the *defb* gene isoforms of olive flounder fish are highly expressed in the testis and during embryonic development, respectively [13], [17]. Therefore, fish defensins are thought to play an important part in the integrity of the reproductive system, perhaps linked to immune defence. The presence of cod *defb* transcripts along the egg membrane of early vitellogenic stage oocytes indicate that Defb may be involved in oocyte maturation.

Exposure of Atlantic cod to live pathogenic bacteria (*V. anguillarum*) was found to induce a 30.6-fold up-regulation of *defb* expression in the head kidney of challenged fish at 48 h post-stimulation. Such an induction was not observed in the mucosal tissues even though the fish were directly exposed to the bacteria. This implies that the head kidney of cod is involved in the recruitment of beta-defensin producing cells in response to bacterial infection. In rainbow trout, upon *Yersinia ruckeri* infection, the *defb* isoforms were differentially expressed by immune tissues and organs [9]. A similar trend was seen in the head kidney of olive flounder after infecting the fish with bacteria, *Edwardsiella tarda* [13], and inducible expression of the rainbow trout beta-defensin gene was observed in *defb1*-transfected EPC cells exposed to viral haemorrhagic septicaemia virus [10]. Furthermore, in the rainbow trout head kidney all four *defb* genes transcripts were seen very clearly after the cell was stimulated with PolyI:C [9]. The prompt up-regulation of cod *defb* upon antigenic challenge strongly suggests that it is involved in the innate immune response. On the other hand, the constitutive expression of *defb* gene found in several tissues of naïve cod may be signalling its role in homeostasis.

The antibacterial spectrum of fish defensins varies significantly amongst different species [11], [13], [14], [17], [44]. Recombinant cod Defb displayed potent antibacterial activity in the micromolar range against Gram-(+) bacteria, namely *P. citreus* and *M. luteus*. A similar selective activity has been reported for gilthead seabream Defb, which is antibacterial against *Bacillus subtilis* but shows little or no activity against the Gram-(**−**) strains *Photobacterium damselae subsp.piscicida*, *Vibrio harvey* and *V. anguillarum* [14]. In contrast, medaka Defb is more active against Gram-(**−**) bacteria even if it is able to inhibit growth of the Gram-(+) bacterium *M. luteus* at similar concentrations to those required by cod Defb [11]. Recombinant Defb from mandarin fish (*Siniperca chuatsi*) seems to display a broad spectrum against Gram-(+) and Gram-(**−**) bacteria but its antibacterial activity has not been quantified [44].

It is noteworthy that cod Defb did not display antibacterial activity against *V. anguillarum*, in spite of marked up-regulation of this gene in head kidney *in vivo* following antigenic challenge with these bacteria. Nevertheless, cod Defb increased phagocytic activity of head kidney leucocytes by over 60% at a concentration of 20 µM. To the best of our knowledge, this is the first report describing phagocytosis stimulation by a beta-defensin but alpha-defensins in human neutrophils are known stimulants of phagocytosis by neutrophils [3]. Also, seabream head-kidney leucocytes showed chemotactic activity towards supernatants containing recombinant Defb [14]. It is plausible that cod *defb* is induced by exposure to *V. anguillarum* because it stimulates phagocytic activity of head kidney leucocytes as an important component of the immune response.

In conclusion, cod *defb* is similar to other known fish beta-defensins in terms of genomic organisation, primary and putative tertiary structures. Expression analysis indicated that cod *defb* gene transcript levels were relatively high in the swim bladder, eyes and oocytes and moderate in skin and head kidney. Recombinant Defb was active only against Gram-(+) bacteria and showed potent stimulatory activity of phagocytosis in head kidney leucocytes. *Defb* was up-regulated *in vivo* 48 h after challenge with pathogenic Gram-(**−**) bacteria. Taken together, our data indicate that cod *defb* may play an important role in the innate immune response.

## Supporting Information

Figure S1 Purification of recombinant cod beta-defensin. Lane 1; non-induced host cell lysate, lane 2; induced host cell lysate, lane 3; BugBuster soluble fraction, lane 4; ProBond purified fraction, lane 5; enterokinase digest and lane 6; purified recombinant cod beta-defensin. Lane M; molecular weight markers.
(TIF)

Table S1 Matches for unspecific trypsin cleavage of cod defensin. Positions of peptide fragments and their monoisotopic masses are indicated. The identified fragments covered the entire cod defensin sequence (FPWSCPTLSG VCRKVCLPTE MFFGPLGCGK EFQCCVSHFF).
(DOCX)
