# Supplementary figures and images for: A Novel Beta-Defensin Antimicrobial Peptide in Atlantic Cod with Stimulatory Effect on Phagocytic Activity
Source: PLoS One. 2013 Apr 25;8(4):e62302. doi: 10.1371/journal.pone.0062302 (PMC3636224; doi:10.1371/journal.pone.0062302)

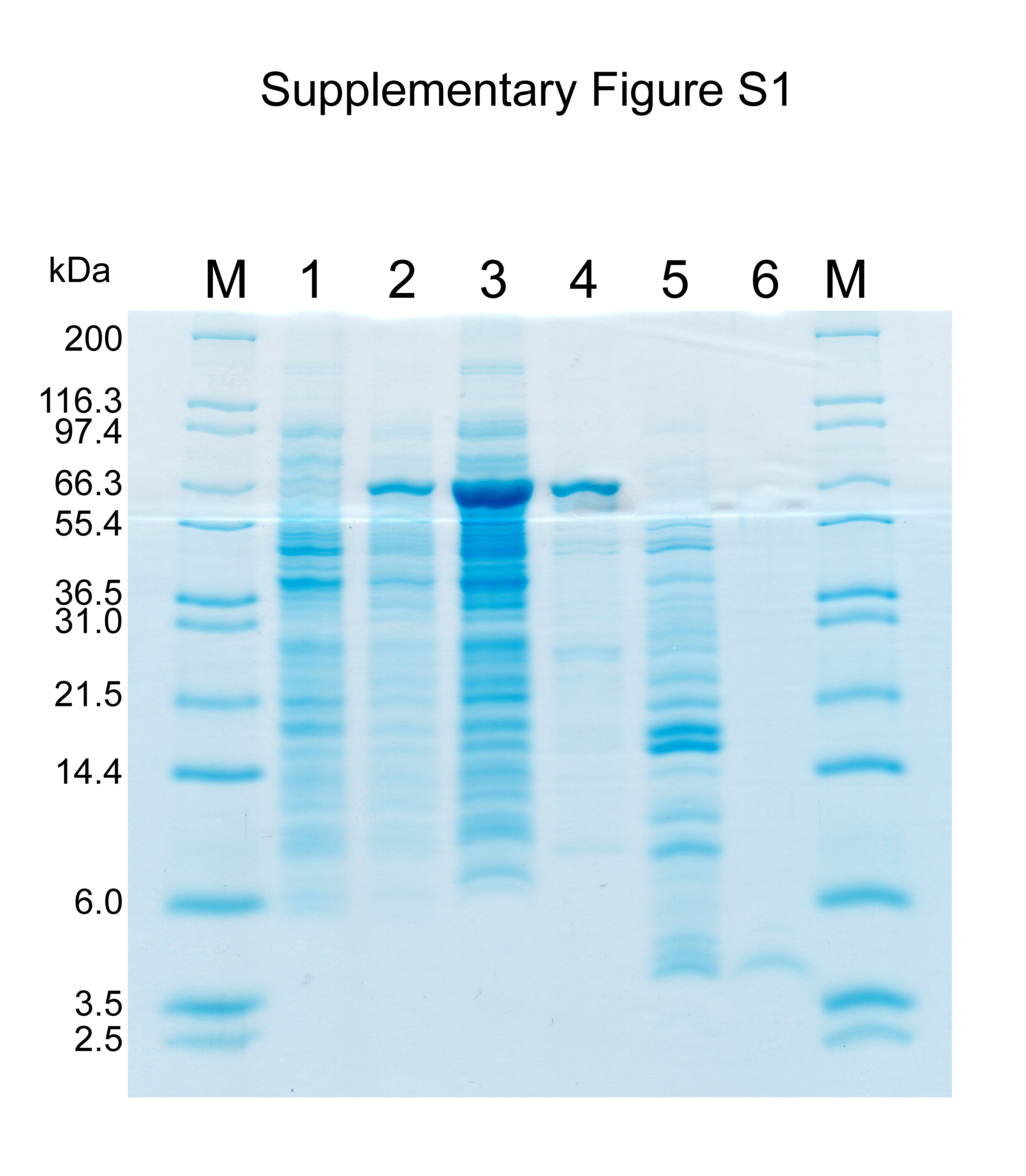

Supplement: Figure S1 — Purification of recombinant cod beta-defensin. Lane 1; non-induced host cell lysate, lane 2; induced host cell lysate, lane 3; BugBuster soluble fraction, lane 4; ProBond purified fraction, lane 5; enterokinase digest and lane 6; purified recombinant cod beta-defensin. Lane M; molecular weight markers. (TIF) [file pone.0062302.s001.tif]
