# Supplementary material for: A Novel Beta-Defensin Antimicrobial Peptide in Atlantic Cod with Stimulatory Effect on Phagocytic Activity
Source: PLoS One. 2013 Apr 25;8(4):e62302. doi: 10.1371/journal.pone.0062302 (PMC3636224; doi:10.1371/journal.pone.0062302)
Supplement: Table S1 — Matches for unspecific trypsin cleavage of cod defensin. Positions of peptide fragments and their monoisotopic masses are indicated. The identified fragments covered the entire cod defensin sequence (FPWSCPTLSG VCRKVCLPTE MFFGPLGCGK EFQCCVSHFF). (DOCX) [file pone.0062302.s002.docx]

**Supplementary table S1**. Matches for unspecific trypsin cleavage of cod defensin. Positions of peptide fragments and their monoisotopic masses are indicated. The identified fragments covered the entire cod defensin sequence (FPWSCPTLSG VCRKVCLPTE MFFGPLGCGK EFQCCVSHFF).

| Peptide sequence | Position | Experimental  mass (Da) | Theoretical  mass (Da) | Δmass  (Da) |
| --- | --- | --- | --- | --- |
| FPWSCP | 1-6 | 793.334 | 793.334 | 0.000 |
| TLSGVCR | 7-13 | 792.432 | 792.403 | -0.028 |
| KVCLPTEMFFGP | 14-25 | 1425.684 | 1425.690 | 0.006 |
| LGCGKEFQC | 26-34 | 1098.483 | 1098.471 | -0.012 |
| CVSHFF | 35-40 | 796.363 | 796.345 | -0.018 |
